# Variant CAPS in an adult- the use of genetics

**DOI:** 10.1186/1546-0096-13-S1-P27

**Published:** 2015-09-28

**Authors:** M Rozenbam, D Rimar, L Kaly, G Slobodin, A Awisat, I Rosner

**Affiliations:** 1 Bnai-Zion medical center, Rheumatology, Haifa, Israel

## Introduction

Cryopyrin-associated periodic syndrome (CAPS) includes three overlapping disorders: familial cold autoinflammatory syndrome (FCAS), Muckle-Wells syndrome (MWS) and neonatal onset multisystem inflammatory disorder (NOMID). Once considerate separate entities, these hereditary autoinflammatory disorders have been found to share a common genetic basis, pathogenesis and treatment and are therefore now considered a continuous clinical spectrum of a single entity. CAPS is caused by dominantly inherited or de novo NRLP3 mutations

## Objectives

The use of genetic methods for detecting somatic NLRP3 mosaicism in adult onset CAPS phenotype

## Patients & methods

A 53 year old male, Christian Arab from North Israel, suffered for 2 years from an unclassified multi-system inflammatory disorder. Its chief features were weight loss, fever, maculopapular skin eruption, arthritis, lymph node enlargement, hepatosplenomegaly, bone periosteal reaction, all associated with a major acute phase response. The arthritis was chronic, with disease flares lasting weeks to months. He responded moderately well to high dose corticosteroids but not to immunosuppressive agents including DMARDs, anti TNF agents, rituximab, nor tocilizumab. Subsequently, he developed a moto-sensory peripheral neuropathy of the legs and sensorineural deafness. There was no evidence of amyloidosis nor a relevant family history.

## Results

No mutations in MEFV, nor for TRAPS and MVK were found.

A variant in NRLP3 Tyr 570 Cys (Y750C) was found in peripheral blood cells and buccal mucosa.

The patient was treated with anti-IL-1 drugs. Anakinra was poorly tolerated and then switched to canakinumab 300 mg / 4 weeks sc with complete remission of all symptoms except for the sensorineural deafness and peripheral neuropathy.

## Conclusion

The detection of somatic mosaicism can have major clinical implications for patients including access to efficacious treatment duly recognized by regulators, and more availability of needed frequent monitoring. Taking into account the patient's excellent response to IL -1 blockade, it is reasonable to hypothesize that its earlier institution might have prevented the appearance of the severe complications of deafness and neuropathy.

